# Vaccinia Virus Infection & Temporal Analysis of Virus Gene Expression: Part 1

**DOI:** 10.3791/1168

**Published:** 2009-04-08

**Authors:** Judy Yen, Ron Golan, Kathleen Rubins

**Affiliations:** Whitehead Institute for Biomedical Research, MIT - Massachusetts Institute of Technology

## Abstract

The family *Poxviridae* consists of large double-stranded DNA containing viruses that replicate exclusively in the cytoplasm of infected cells.  Members of the *orthopox* genus include variola, the causative agent of human small pox, monkeypox, and vaccinia (VAC), the prototypic member of the virus family.  Within the relatively large (~ 200 kb) vaccinia genome, three classes of genes are encoded: early, intermediate, and late.  While all three classes are transcribed by virally-encoded RNA polymerases, each class serves a different function in the life cycle of the virus.  Poxviruses utilize multiple strategies for modulation of the host cellular environment during infection. In order to understand regulation of both host and virus gene expression, we have utilized genome-wide approaches to analyze transcript abundance from both virus and host cells.  Here, we demonstrate time course infections of HeLa cells with Vaccinia virus and sampling RNA at several time points post-infection.  Both host and viral total RNA is isolated and amplified for hybridization to microarrays for analysis of gene expression.

**Figure:**
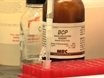


## Protocol

### Part 1: Setting up the infection

1. Grow HeLa cells in flasks and wait until the cells are approximately 80% confluent.
2. Prepare enough viral growth medium for the experiment: regular DMEM with 2% FBS and no added antibiotics.
3. The infection can be performed with either a crude stock of vaccinia virus with a known titer, or sucrose purified virus with a known titer if you're concerned about host gene expression.
4. If you are using sucrose purified virus, proceed directly to part 2.
5. If you are using a crude vaccinia virus stock, just prior to the infection, sonicate an aliquot of the virus in a cup sonicator with a bath of mostly ice and a little water.
  - Sonicate at around 30W for 20 seconds.
  - Vortex the tube and repeat the sonication 3 times, adding more ice if needed to keep the cup packed with ice and chilled.
  - Vortex in between each sonication step.
  - Proceed to part 2.
6. Alternatively, if you do not possess a cup sonicator, mix an equal volume of the crude virus stock and 0.25mg/mL trypsin.
  - Vortex vigorously.
  - Incubate the virus stock/trypsin mix in a 37°C waterbath for 30 minutes.
  - Vortex at 5-10 minute intervals.
  - Proceed to part 2.

### Part 2: Infecting cells

1. Calculate the amount of viral particles needed to infect the monolayer at the desired multiplicity of infection (MOI).  Typically, a high MOI (between 5 and 10) is used for infection timecourses.
2. Add the desired amount of sucrose purified virus or trypsinized crude virus to 37°C viral growth media and mix well.  You should use enough media to just cover the bottom of the flask.  For example, 10 mL of media for a T-175 flask.
3. Remove media from the cells and rinse with room temperature PBS.
4. Add the virus/viral growth media to each flask.
  - Swirl gently, tilt plates and incubate at 37°C in a 5% CO2 incubator for 1 hour.
  - Tilt and swirl plates every 15 minutes to spread virus uniformly and keep cells moist.
  - For a 0hr/Mock time point, add the viral growth media only, with no virus added.
5. After the 1 hour incubation, remove the media containing the virus, and rinse three times with room temperature PBS.
6. Add the maximum amount of viral growth medium to each flask.  For example, 30 mL of media for a T-175 flask.  Start counting this as your 0 hr time point.

### Part 3: Harvesting cells

1. Check the cells under a microscope and note any cytopathic effect (CPE).
2. Remove the media and rinse cells with 30 mL of room temperature PBS.
3. Add 15 mL of trypsin to the cells and incubate for 2-5min at 37°C.
4. Check under microscope for cell detachment and tap flask gently to dislodge cells.
5. Transfer cells with a sterile serological pipette into a 50 mL conical tube.
6. Wash the flask with an equal volume of cell growth media, and add the media to the trypsinized cells in the conical tube.
7. Centrifuge the cells at 300 x *g* for 5 minutes at room temperature.
8. Remove the trypsin/media from the cell pellet.
9. Resuspend the cell pellet in either TRIzol reagent or the lysis buffer from your desired RNA isolation kit.
10. If you are using TRIzol, divide the sample into 1 mL aliquots in 1.5mL labeled Eppendorf tubes and freeze at -80°C.
11. Repeat for all time points, harvesting one flask per time point.

### Part 4: RNA extraction of samples in TRIzol

1. At this point, your samples should be resuspended in TRIzol reagent and ready to be processed.
2. Add 200µL of the BCP Phase Separation Reagent for every 1 mL of TRIzol in each tube.  You may need to transfer the sample to a larger tube if you are starting out with a large amount of TRIzol.
3. Vortex or shake vigorously and incubate at room temperature for 2-3 minutes.
4. Centrifuge the sample at 12,000 x g for 15 minutes.
5. Transfer the aqueous phase to a fresh tube.  The aqueous phase is the clear layer on the top. You should be recovering approximately 600µL for every 1mL of TRIzol you started out with.
6. Do a second phenol/chloroform extraction, this time using 500µL of chloroform per 1 ml TRIzol, instead of BCP.
7. Vortex or shake vigorously and incubate at room temperature for 2-3 minutes.
8. Centrifuge the sample at 12,000 x g for 15 minutes.
9. Transfer the aqueous phase to a fresh tube.  The aqueous phase is the clear layer on the top.
10. Add 20µg of linear acrylamide to each sample.  The linear acrylamide acts as a carrier and helps to precipitate the RNA.
11. Add 500µL of isopropanol per 1 ml of TRIzol you started out with to each sample and mix well.
12. Incubate samples at room temperature for 10 minutes.
13. Centrifuge in a microcentrifuge at maximum speed for 10-15 minutes.
14. The RNA pellet will be visible at the bottom of the tube.  Very carefully, remove the supernatant from the tube, either with a vacuum aspirator, or manually by pipettor.  Do not disturb the pellet.
15. Wash the pellet with 1 mL of 70% ethanol.
  - Add 1 mL of 70% ethanol to the pellet.
  - Centrifuge at top speed 14,000g for 5-7 minutes.
  - Very carefully, remove the ethanol from the tube, either with a vacuum aspirator, or manually by pipettor.  Check that the pellet is visible at the bottom of the tube.
  - Discard the supernatant.
16. Repeat the wash step.  After the supernatant is discarded, mark where pellet is on the tube.
17. Air dry pellet for no longer than 5 minutes.  Do not overdry, or the RNA will be difficult to reconstitute!
18. If you wish to follow the optional DNase treatment to remove any remaining DNA from the sample, resuspend the pellet in 17µL of nuclease-free water.  Otherwise, resuspend the pellet in 20µL of nuclease-free water and continue to step 22.
19. (Optional DNase treatment) Using the Qiagen RNase-free DNase set, add 2µL Buffer RDD and 1µL reconstituted RNase-free DNase I to the resuspended RNA.  Mix gently.
20. (Optional DNase treatment) Incubate at 37°C for 30 minutes.
21. (Optional DNase treatment) Add 2µL of 2.5 mM EDTA and incubate at 65°C for 5 minutes to inactivate the DNase.  Do not exceed the inactivation time or temperature as longer times/higher temperatures can cause degradation of the RNA.
22. Check the RNA concentration by spectrophotometer.
23. Store the RNA sample at -80°C.
24. (Optional QC) Check the RNA quality using an Agilent BioAnalyzer, or by running the sample on a denaturing gel and checking the O.D. readings. (260nm and 260/280 ratio)

## Discussion

### Critical Steps

#### Part 1 & 2

There are several critical steps to setting up a synchronous vaccinia infection, the first being careful sonication (or trypzinizing) of the virus, in order to disaggregate virus particles.  Vaccinia is highly prone to aggregating, and disruption of virus particles is important for ensuring even infection of cells.  In order to achieve a synchronous infection, a high MOI (greater than 2) should be used to ensure each cell is infected.  A mixture of infected and uninfected cells will lead to multiple rounds of infection, heterogeneous mixtures of time points, and asynchronous viral and host transcriptional responses.  The infection should be carried out in minimal amounts of media to allow maximum adsorption of virus onto the cells.  In addition, regular shaking of the flasks or culture dishes (every 10 minutes) enables distribution of virus across the flask and ensures that the cells do not dry out.

#### Part 4

1-Bromo-3-chloropropane (BCP) is used in place of phenol to reduce genomic DNA contamination.  A subsequent optional DNAse treatment (Qiagen RNAase Free-DNAse) may also be performed to eliminate any remaining genomic DNA.  A second chloroform extraction is used to remove any traces of organic solvent from the extraction, as even trace amounts can inhibit subsequent amplification steps.  Traces of phenol/BCP can be detected as a spike at 270nm (beyond the standard peak at 260nm) when measuring absorbance of total RNA after extraction.  If such contamination appears, re-extract the RNA (using a filter or column-based RNA extraction method) before proceeding to amplification.  Minimum amount of RNA needed to perform an amplification reaction is 100ng; however, 500-1000ng is preferred.

### Application/Significance

The labeled RNA resulting from this protocol can be hybridized to human, viral, or custom microarrays to assess gene expression responses to infected cells in culture.  Microarray platforms vary, so follow manufacturer instructions for preparation of hybridization mixture from labeled probe.

Using a custom designed poxvirus array^1^, we were able to classify genes into the general categories of “early” or “late” based on timing of hybridization signal and whether or not viral DNA replication was required for transcript detection.  We observed the expected functional categories of genes in each temporal class (i.e., expected early, intermediate and late genes) variation as to the exact timing of transcription.

The methods utilized in this work are able to predict virus genes transcribed early or late in the replication cycle, but have more difficulty distinguishing early-only versus genes with an early and late promoter since transcripts with a dual early/late promoter may persist and be detected at late times. In addition, run-through transcription of late viral genes may affect signal at a given probe/spot on the array, as the RNA hybridizing to the array may have come from the designated ORF or an upstream ORF.  Tiling arrays have attempted to resolve this issue, however challenges remain in detecting run through transcription using hybridization based approaches^2,3,4^.

Host transcriptional patterns can also be assessed using these methods.  However, vaccinia encodes a variety of mechanisms to inhibit host responses, and host transcriptional responses may be diminished compared to other stimuli^5,6,7,8^.  Since the expression of many genes involved in host defense is altered after infection, the contribution of viral genes that counteract host immune responses should therefore be taken into consideration.

Utilizing these methods, a map of the transcriptional timing of all viral genes can be identified and used to interrogate functions of unknown viral genes.  In addition, these methods can be utilized to dissect the intricate dialogue between virus and host.  These methods are broadly applicable to other host-pathogen infection systems.  If the pathogen of interest does not have polyadenylated mRNAs, alternative methods can be used to directly label the total RNA, without linear amplification.  By analyzing both host and virus gene expression during synchronous infection, these methods allow us to gain insight into virus interaction with the host cellular environment as well as host counter-defenses against virus infection.
